# Interactions between reptiles and people: a perspective from wildlife rehabilitation records

**DOI:** 10.1098/rsos.240512

**Published:** 2024-10-02

**Authors:** Teagan Pyne, Ron Haering, Aditi Sriram, Shona Lorigan, Richard Shine, Chris J. Jolly

**Affiliations:** ^1^ School of Natural Sciences, Macquarie University, New South Wales 2109, Australia; ^2^ Department of Climate Change, Energy, the Environment and Water, NSW Government, Parramatta, New South Wales 2150, Australia; ^3^ Research Institute for the Environment and Livelihoods, Charles Darwin University, Casuarina, Northern Territory 0810, Australia

**Keywords:** conservation, lizard, Reptilia, rescue, snake, Squamata

## Abstract

As urbanization expands globally, human–wildlife interactions will inevitably increase. Here, we analysed 10 years of wildlife rehabilitation records of squamate (snake and lizard) reptiles (*n* = 37 075) from the Greater Sydney region, New South Wales, Australia, to explore their value to address management and conservation issues. Rescues were highly non-random regarding taxonomic focus, spatial occurrences and temporal trends due to the combined influence of (i) reptile phenology and behaviour and (ii) human perceptions of reptiles. Seasonal peaks in rescues reflect reptile and, to a lesser extent, human activity. Spatial patterns of rescues were informative about distributions and presence of easily identified taxa but were primarily driven by human presence. Larger squamate species were rescued more frequently, potentially reflecting a perception of greater danger or rescue priority. While uncommon species were often misidentified, accurate reports of these taxa may guide targeted surveys. The value of these data for conservation and management could be enhanced by emphasizing reptile identification training of volunteers and use of applications for informed species identification. Wildlife rehabilitation data offer a cost-effective means of quantifying thousands of human–reptile interactions, identifying foci (in both time and space) of human–wildlife conflict such as snakebite risk and roadkill-related reptile mortality.

## 1. Introduction

Conservation and management rely on accurate, relevant and reproducible data [1]. Traditionally, information about wildlife distribution and abundance is gathered by trained professionals, using quantitative surveys [2]. However, such methods are resource-intensive and time-consuming [1], leading biodiversity managers to look elsewhere for data. Citizen science is increasingly being employed for that purpose [3,4], often based on unstructured projects with broad objectives and minimally trained observers [2,4]. Technologies such as mobile phone applications and document clouds have facilitated the capture and storage of vast repositories of biological information by the public [1]. An activity with a long history and involving many people is the collection of wildlife rescue data by volunteer wildlife rehabilitation organizations [5–11]. This activity generates large, long-term datasets, which are readily accessible and regularly updated.

**Figure 1.**
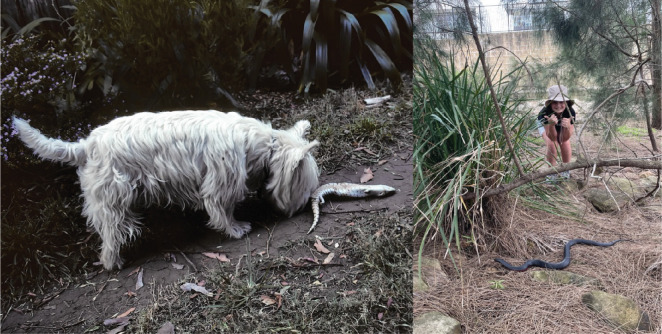
Squamate reptiles in urban Sydney, New South Wales, Australia. Urban reptiles, such as this eastern blue-tongue lizard (*Tiliqua scincoides*), face the novel threats of pet attack and car strike and may require relocation or rehabilitation. Large venomous elapid snakes, such as this red-bellied black snake (*Pseudechis porphyriacus*), are found in urban areas of Sydney and are often perceived as a threat to humans and pets. Photos supplied by Richard Shine and Matthew Greenlees.

Urbanization has increased human–wildlife interactions and, in turn, the number of reports made to wildlife rehabilitation organizations [12]. Wildlife in urban areas encounter threats such as vehicle strikes, pollution and attacks by domestic pets [13–16] (figure 1). Although such interactions are often negative [17], not all human–wildlife interactions are harmful for wildlife. For example, generalist snake species like carpet pythons (*Morelia spilota*) are able to thrive in urban conditions [18,19]. To understand both the positive and negative effects of urban development on native fauna, we need to document the distribution and detection frequency of wildlife in these settings.

The rescue, rehabilitation and release of sick, injured or orphaned native animals are common practice in Australia. There are more than 20 000 registered wildlife rehabilitation volunteers across the country [20], but with varying levels of training and experience. In New South Wales (NSW), Australia, there are over 8600 volunteers who are members of 41 wildlife rehabilitation providers [21]. These volunteers respond to more than 200 000 wildlife rescue assistance calls from the public and attend over 110 000 rescues annually [21]. All wildlife rehabilitation providers are licenced under the NSW *Biodiversity Conservation Act 2016* (BC Act) and are regulated by the NSW National Parks and Wildlife Service (NPWS). Licence holders are required to comply with quality assurance requirements including Codes of Practice which specify minimum standards for animal welfare, training of members and the keeping and mandatory submission of records [6]. Licence holders are also required to collect and report wildlife rescue data on an annual basis. Volunteers collect data on the species, reason for rescue, location and outcome of rescue. Several decades of wildlife rescue data are available, with the first and largest wildlife rehabilitation organization in NSW, Wildlife Information, Rescue and Education Services (WIRES) established in 1985 [15]. However, biases around the use of wildlife rehabilitation data have discouraged the use of this information by researchers. For example, misidentifications are common, and concentration of effort on specific taxa may be driven by human behaviour rather than wildlife abundance [22].

Charismatic native animals receive greater conservation effort [23,24]. This pattern is evident from the number of mammal, bird and reptile rescues reported in NSW. Between 2013 and 2018 in NSW, mammals and birds comprised 34.1% and 53.4% of rescues, respectively, while reptiles only accounted for 12.5% of rescues [15]. While some species of reptiles are more cryptic than some birds and mammals in urban areas, such results are also consistent with studies showing reptiles are unpopular with the public and are often perceived as dangerous or ‘disgusting’ [23–26]. Indeed, fear of snakebite is one of the most common reasons why reptiles are ‘rescued’ [27].

Using data collected by 17 NSW wildlife rehabilitation providers between 2011 and 2021, we have explored five issues:

— What species of squamate reptiles (i.e., lizards and snakes) are rescued most often?
— Do frequently rescued species exhibit distinctive characteristics such as large body size?
— Has the species composition of reptile rescues changed since a previous assessment of data collected in the same area 20 years previously [28]?
— How common are species misidentifications?
— How and why do rescue rates vary through time (seasonally) and space (distribution)?

## 2. Methods

### 2.1. Study area

**Figure 2.**
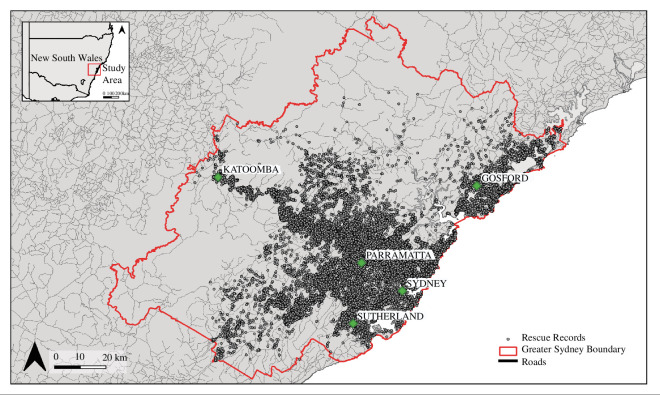
Distribution of snake and lizard records from rescues performed in the Greater Sydney region between 2011 and 2021 (*n* = 37 075). Inset map shows the study area in the context of NSW, Australia.

Greater Sydney, NSW, covers approximately 550 km^2^ in south-eastern Australia. We define ‘Greater Sydney’ to include all of metropolitan Sydney and surrounding areas, plus the local government areas of the Central Coast, Hawkesbury, Blue Mountains, Wollondilly and Sutherland Shire [29] (figure 2). The region is highly urbanized and experiencing rapid urban expansion [13,30]. Urban areas are interspersed with green spaces such as parks and gardens, as well as national parks.

### 2.2. Data collection

Data analysed in this study were acquired from 17 wildlife rehabilitation providers who operate throughout Greater Sydney. Pursuant to their Biodiversity Conservation licence, these organizations can perform rescue and rehabilitation activities including possession, release and harm (i.e. pursue, capture and/or euthanasia). Wildlife rehabilitators are required to submit data for each animal callout they attend and provide information on the rescued animal’s species, age and sex, as well as the date, location, reason for rescue, animal condition and outcome (e.g. release date or euthanasia) [31]. These data are entered into a publicly accessible spreadsheet template [32] and submitted annually.

### 2.3. Data preparation and filtering

Records for the Greater Sydney region were extracted from a larger NSW-wide dataset assembled by the NSW NPWS, covering a period from 1 January 2011 to 31 December 2021. From a total of 60 398 callout records, data were filtered to remove records with incomplete information (e.g. no species name). We removed marine and non-squamate reptiles (e.g. sea turtles, freshwater turtles and crocodiles). We also removed records for species not found in the Greater Sydney region [33] and not easily assigned as a misidentified local species (see electronic supplementary material, table S1), on the basis that these were either unresolvable misidentifications or escaped pets. The only exception to this was the inclusion of shingleback lizards (*Tiliqua rugosa*), given the high frequency with which this morphologically unique (and thus, easily identified) taxon was recorded. Taxa were classified to species level. Following this data filtering, we retained 37 075 records.

As a measure of body size for our analyses, we used information on snout-vent lengths (SVL) for species from Shine [34] and Wilson and Swan [33]. For conservation status, we used listings under the BC Act, the *Environmental Protection and Biodiversity Conservation Act 1999* (EPBC Act) and the International Union for Conservation of Nature’s Red List of Threatened Species.

### 2.4. Data analysis

The record distribution map was developed using ArcGIS Pro 3.1.3, by overlaying the coordinate data over a map of Greater Sydney. Visual comparisons of body size distributions were made between (i) all lizard species in the Greater Sydney region, (ii) lizards that were rescued, and (iii) the species most encountered during rescues. The same comparisons were performed for snake species. Because these data were not normally distributed, we used Spearman’s rank correlation to assess the relationship between maximum SVL for the species of lizards and snakes that were rescued and the number of times they were rescued. We performed a linear regression to compare the numbers of the eight squamate species reported to have been rescued between 1989 and 1998 by Shine and Koenig [28] and the number of these species rescued in the present study. These analyses were performed using R version 4.3.2 [35]. In all other cases, we did not use statistical tests to evaluate the ‘significance’ of the resulting patterns, because the massive sample sizes render even a small (and perhaps biologically unimportant) difference significant in such a test.

## 3. Results

### 3.1. What species of squamate reptiles are rescued most often?

Between 2011 and 2021, >37 075 squamate reptiles were rescued by 17 wildlife rehabilitation providers in the Greater Sydney region (electronic supplementary material, table S1). Most records were attributed to WIRES, with 72.27% (*n* = 26 795) of records coming from this organization. Rescued taxa were identified to 53 species representing 10 families, a substantial proportion of the 62 squamate species from 10 families that occur naturally in the study area (electronic supplementary material, table S2). However, rescue efforts were dominated by a small subset of species. Of the total records, 98.73% (*n* = 36 604) involved only 20 taxa (table 1). Two species, the red-bellied black snake (*Pseudechis porphyriacus*) and the eastern blue-tongue lizard (*T. scincoides*), comprised almost two-thirds of all records (*n* = 24 553, 66.23%). The most common reason for rescue was ‘unknown’ (*n* = 28 122, 75.85%), followed by ‘unsuitable environment’ (*n* = 4222, 11.39%) and ‘attack’ (*n* = 2348, 6.33%) (see electronic supplementary material, table S3, for definitions of encounter types; electronic supplementary material, table S4). The most frequently reported animal fate was ‘released/relocated’ (*n =* 10 770, 29.05%), followed by ‘euthanized or died in care’ (*n =* 6407, 17.28%) (see electronic supplementary material, table S3, for definitions of animal fates; electronic supplementary material, table S5). Four correctly identified species (*n* = 20 individuals) listed as threatened were reported to have been rescued (electronic supplementary material, table S1).

**Table 1.** The 20 most common species of squamate reptiles recorded by wildlife rehabilitation providers between 2011 and 2021. These records comprise 98.73% of all records in the overall dataset (*n* = 37 075). ^a^Marked species indicate taxa that are not native to the Greater Sydney region (and hence likely are misidentifications or escaped pets).

sub-order	species	count
lizard	eastern blue-tongue (*Tiliqua scincoides*)	14 515
snake	red-bellied black snake (*Pseudechis porphyriacus*)	10 038
snake	carpet python (*Morelia spilota*)	2123
lizard	water dragon (*Intellagama lesueurii*)	2041
snake	eastern brown snake (*Pseudonaja textilis*)	2000
snake	green tree snake (*Dendrelaphis punctulatus*)	1819
lizard	lace monitor (*Varanus varius*)	1142
snake	golden-crowned snake (*Cacophis squamulosus*)	764
lizard	eastern bearded dragon (*Pogona barbata*)	466
snake	yellow-faced whipsnake (*Demansia psammophis*)	253
snake	tiger snake (*Notechis scutatus*)	247
lizard	sand goanna (*Varanus gouldii*)^a^	245
snake	marsh snake (*Hemiaspis signata*)	181
lizard	blotched blue-tongue (*Tiliqua nigrolutea*)	170
snake	common death adder (*Acanthophis antarcticus*)	169
snake	highland copperhead (*Austrelaps ramsayi*)	155
snake	brown tree snake (*Boiga irregularis*)	84
lizard	eastern water skink (*Eulamprus quoyii*)	68
lizard	shingleback (*Tiliqua rugosa*)^a^	65
lizard	southern leaf-tailed gecko (*Saltuarius swaini*)^a^	59

### 3.2. Do frequently rescued species exhibit distinctive characteristics such as large body size?

**Figure 3.**
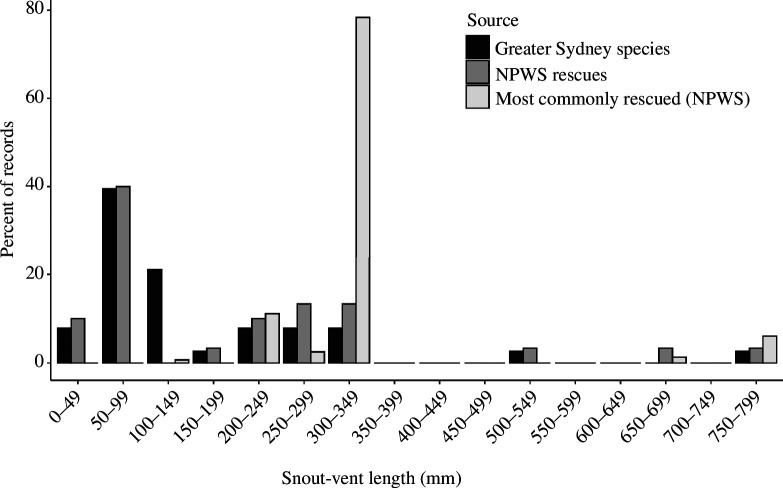
Frequency distributions for mean body sizes (snout-vent lengths = SVLs) of lizard species (*a*) found naturally within the Greater Sydney region (‘Greater Sydney species’, black bars), (*b*) represented in the rescue dataset (i.e. recorded at least once; ‘NPWS rescues’, dark grey bars) and (*c*) belonging to the 10 most commonly rescued species (‘most commonly rescued (NPWS)’, light grey bars).

**Figure 4.**
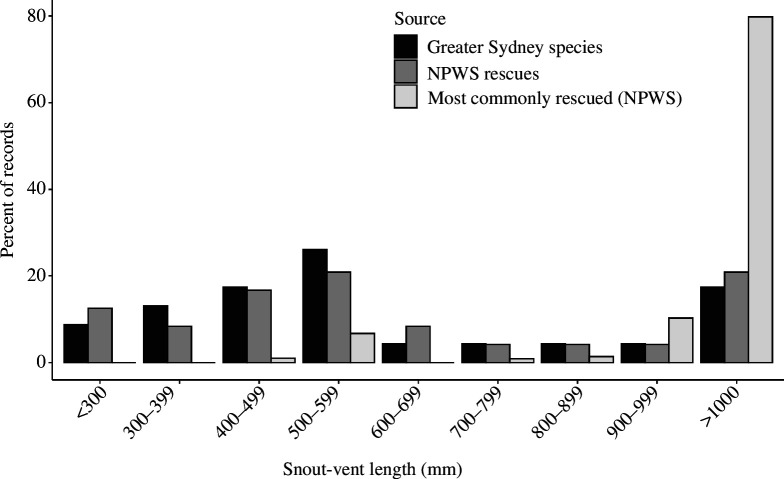
Frequency distributions for mean body sizes (snout-vent lengths = SVLs) of snake species (*a*) found naturally within the Greater Sydney region (‘Greater Sydney species’, black bars), (*b*) represented in the rescue dataset (i.e. recorded at least once; ‘NPWS rescues’, dark grey bars) and (*c*) belonging to the 10 most commonly rescued species (‘most commonly rescued (NPWS)’, light grey bars).

The rescue data show a pronounced bias towards larger reptiles, with a significant positive correlation between snake and lizard SVL and number of rescues (snakes: ρ = 0.54, *n* = 23, *p* = 0.008; lizards: ρ = 0.75, *n* = 30, *p *< 0.001). Most lizard species known to occur in the Greater Sydney region are small (<100 mm; see figure 3), whereas the species most commonly rescued were large (modal peak = SVL of 300−349 mm; see figure 3). This discrepancy largely reflects the numerous records of eastern blue-tongues, as well as two similarly sized congeners (blotched blue-tongue *T. nigrolutea*; shingleback *T. rugosa*). A similar trend was observed for snakes; most species naturally occurring in the study area are small, but most rescues are of large species (SVL > 1000 mm: figure 4), especially red-bellied black snakes. More generally, a few large species dominated rescue numbers, despite this size class containing less than 20% of all snake species in Sydney [34].

### 3.3. Has the species composition of rescues changed since an assessment conducted in the same area 20 years previously?

**Figure 5.**
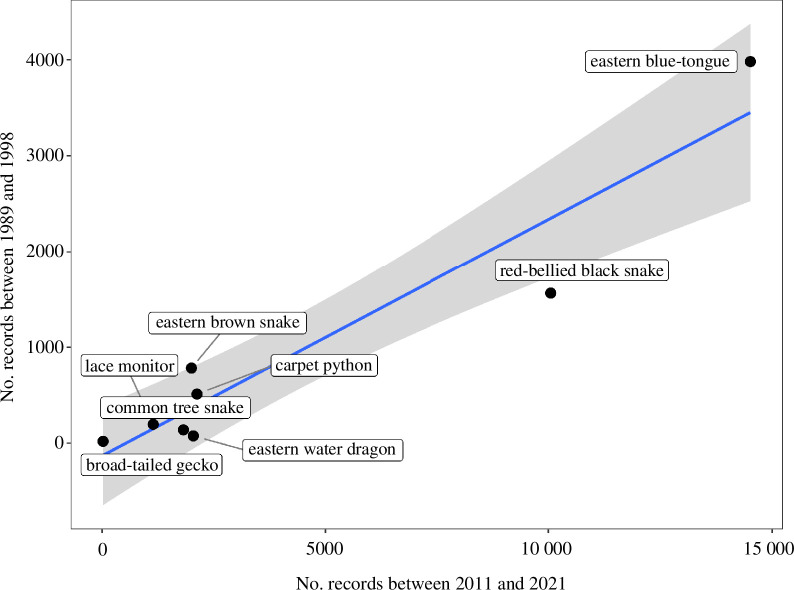
A comparison between the numbers of reptile rescues by wildlife rehabilitation providers in Greater Sydney between 1989 and 1998 [28] and the number of those species rescued between 2011–2021 (present study). The trend line depicts the linear regression between the number of rescues of each species between the two datasets with a 95% confidence interval.

Shine and Koenig [28] analysed 7266 rescues of squamate reptiles from the Greater Sydney area, from data collected over the period between 1989 and 1998. They reported eight species of squamate reptiles rescued in Sydney, all of them relatively large-bodied. Despite the much larger sample size from the current dataset, the numbers of each of those eight species recorded in the 2001 study are very highly correlated with the numbers recorded in the current analysis (see figure 5; *r* ^2^ = 0.90, *F*_1,6_ = 55.76, *p *< 0.001). Notably, red-bellied black snakes and eastern blue-tongues have remained by far the most frequently rescued species in the Greater Sydney area for at least 30 years. Interestingly, while the seven most common squamates remained unchanged (with slight variations in order) between 1989−1998 and 2011−2021, there are now 19 species more frequently rescued between the seventh (*Varanus varius*) and eighth (*Phyllurus platurus*) most common squamates rescued according to Shine and Koenig [28] (electronic supplementary material, table S6).

### 3.4. How common are species misidentifications?

We located 805 records across 38 species (approximately 2.10% of the dataset) of species not naturally found in the study area (see electronic supplementary material, table S7). In most cases, the identification likely was an error based on non-local, but morphologically similar species. For example, eight records for the granite thick-tailed gecko (*Uvidicolos sphyurus*) likely reflect misidentification with an unreported local species, the thick-tailed gecko (*Underwoodisaurus milii*). Similarly, 245 records of sand goannas (*Varanus gouldii*)—the fifth most common lizard in the dataset—are almost certainly misidentified lace monitors (*Varanus varius*) and/or heath monitors (*V. rosenbergi*), which have a conservation status listing of ‘Vulnerable’ under the NSW BC Act. Notably, shingleback lizards (*T. rugosa*) were the ninth most frequently rescued lizard species (*n* = 65) although this iconic species and common domestic pet do not occur naturally in the Sydney region.

### 3.5. How and why do rescue rates vary through time (seasonally) and space (distributions)?

**Figure 6.**
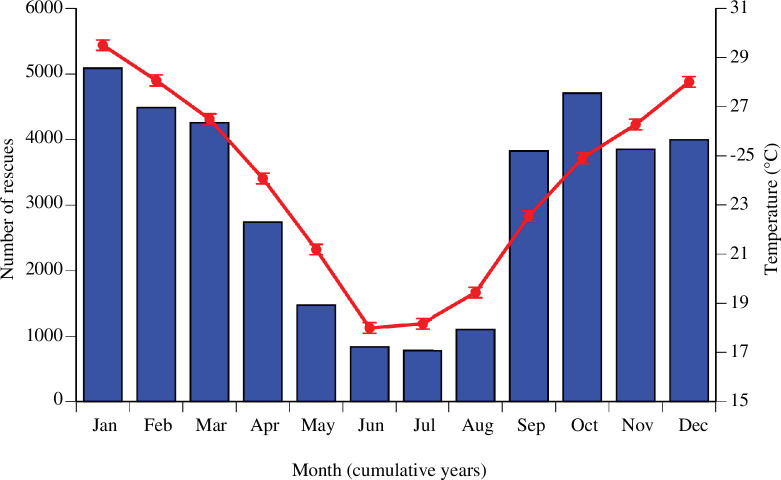
Histogram showing (*a*) the total number of rescues of snakes and lizards per month between 2011 and 2021 and (*b*) mean maximum daily air temperatures per month over the same period, as taken from a Bureau of Meteorology weather station in Parramatta, close to the centre of the area from which reptile-rescue records were obtained.

The data show clear seasonal patterns in rescues (figure 6). Between 2011 and 2021, the number of records per month was greatest during warmer months. The number of records tripled between August and September, with the advent of spring (August: *n* = 1094; September: *n* = 3823). Rescue numbers remained high throughout spring and summer, with midsummer (January) the busiest month (*n* = 5080). A decline then began in April, with the lowest number of records reported in July (*n* = 769).

The spatial distribution of records across the Greater Sydney region was also non-random, with higher rescue rates at some sites than others (figure 2). The highest density of records occurred in metropolitan Sydney and on the Central Coast, with concentrations of rescues distributed along major roads and near national parks. For example, a line of records that extends west out of metropolitan Sydney follows a major highway, the M4 Western Motorway. In essence, the distribution of rescue records mirrors the distribution and density of the human population.

## 4. Discussion

Our analysis emphasizes the magnitude of the wildlife rescue enterprise in modern-day urban Australia, with several thousand reports each year within the Greater Sydney area. Although these numbers are about tenfold higher than in a survey conducted 20 years earlier [28], the same large-bodied species dominate both datasets. Given the high species diversity of squamate reptiles that occur naturally in the study area (62 species), it is a striking fact that two species (one snake, one lizard) constitute almost two-thirds of all reported rescues.

The significance of large body size is apparent not only from the two most frequently rescued taxa but also from all the species ranked below them. For example, lace monitors (*V. varius*) and water dragons (*Intellagama lesueurii*), some of the largest species in their families [33], are commonly rescued. Large elapid snakes are typically uncommon in highly disturbed suburban habitats (e.g. [36]), yet represent a third of all rescues (*n* = 12 462; 33.61%). While larger reptiles may simply be more easily detected because of their relative size, this pattern has likely arisen as people associate large size with a greater threat [24,37]. Thus, smaller reptiles may be perceived as less threatening. The two most abundant reptile species in urban habitats within this region undoubtedly are garden skinks (*Lampropholis delicata and L. guichenoti*: see [38]), which in combination accounted for <0.05% (*n* = 16) of rescue records. Clearly, the numerical composition of rescue records does not reflect the underlying presence of different kinds of reptiles within the study area.

Misidentification can be a major problem also. Most members of the public can likely identify an eastern blue-tongue. However, even distinctive snakes like red-bellied black snakes are often misidentified by the public (e.g. [39,40])—typically, with snakes of smaller dark-coloured taxa being labelled erroneously as ‘red-bellied blacks’ (e.g. eastern small-eyed snakes *Cryptophis nigrescens*). Misidentification increases with smaller species that lack obvious distinguishing characteristics, at least to an untrained eye. NSW wildlife rehabilitation organizations require prospective rescuers to undergo training [31], but the ability to identify taxa to the species-level is not currently a mandatory standard pre-requisite for wildlife rehabilitators. The consequences of this on a day-to-day basis are low; the rescuer’s primary concern is to ensure the threat to humans and animals is resolved while meeting the animal’s welfare needs. However, misidentification reduces the reliability of information in a database. For example, 11 species of gecko were recorded by rescuers, whereas only 4 gecko species are native to the Sydney region [33]. The additional species are predominantly explained by misidentification rather than being escaped or released pets. For example, southern leaf-tailed geckos (*S. swaini*) were more commonly recorded than were broad-tailed geckos (*P. platurus*), despite the former species being naturally restricted to areas near the Queensland border [33]. Additionally, dozens of records also likely involved escaped pets. For example, shinglebacks were frequently rescued but are not native to the Greater Sydney region.

The misidentification of threatened species is particularly concerning as it can influence conservation outcomes. For example, sand goannas (*V. gouldii*) were the fifth most common lizard in the dataset, yet do not occur in the Greater Sydney region. These records likely represent misidentified heath monitors (*V. rosenbergi*)—a vulnerable species that occurs in Sydney but is largely absent from the dataset. Some of these dubious records have already been entered into the nationwide Atlas of Living Australia (ALA) database [41], which is often used as the data source for assessing species geographic distributions, producing species distribution models, designing targeted surveys and developing conservation actions. Erroneous identification of these and other species can weaken our understanding of species distributions, relative abundance and occupancy [42,43], and affect threatened species management plans. It can also result in negative rehabilitation outcomes as reptile care is species specific [44]. As such, caution is needed when using such data to investigate the abundance and distribution of threatened species.

The uncertainties around species identification were difficult to resolve. We attempted to correctly interpret or remove records that included exotic species, escaped pets (except shinglebacks) or misidentified individuals. However, confirmation of the identified species was impossible. In the context of threatened species management, the consequences of contributing inaccurate records to national databases (such as the ALA) could result in important threatened species not being recorded or a population being incorrectly thought to be increasing or declining. A simple solution that would increase the reliability of identification would be to require wildlife rehabilitators to verify records with more experienced wildlife rehabilitators such as their species coordinators or submit a photograph of each rescued animal to a central database. In that way, any dubious or unexpected records could be later vetted by experts. Alternatively, such photos could be filtered through pre-existing social networks that are purpose-built to catalogue and categorize natural history observations, such as iNaturalist [45]. Applications like iNaturalist verify species identifications via crowdsourcing before supplying records to national databases such as ALA. Such vetting of rescue records would dramatically improve the accuracy and efficacy of wildlife rehabilitation data. Encouragingly, new standards for wildlife rehabilitation providers will be introduced in NSW, which will make reptile species identification training mandatory (S Lorigan, personal communication, 2024).

A clear seasonal trend was evident, with records increasing during warmer months. This reflects the seasonal fluctuation in activity observed in squamates along the east coast of Australia [46]. As ambient temperatures increase between September and October, snakes and lizards emerge and may be observed basking in the sunlight or on warmed surfaces. In addition, there is an increase in mate-seeking behaviour during spring, which may explain an increase in the number of injured reptiles during this time [28]. Activity patterns of people, as well as reptiles, likely change seasonally, with outdoor activities such as gardening and bushwalking more popular as winter gives way to spring (e.g. [47]). Regardless of whether the strong seasonal shift in rates of rescue is driven by the seasonality of reptiles or people, the result is an increase in human–wildlife interactions; and the rescue database gives a comprehensive view of where and when such conflicts are likely to occur. As a result, attempts to reduce the frequency of snakebites, or to reduce the toll of motor vehicles on lizards and snakes, could utilize the rescue database to identify when and where such actions would be most effective. For example, such data could be used to inform targeted public education campaigns around seasonal snake activity; this could have a positive effect on reducing potentially negative human–wildlife encounters.

The spatial distribution of rescue records reflects the density of human populations in the Greater Sydney region. This is unsurprising; the public makes these reports, so places with more people report more human–wildlife interactions. Similarly, Taylor-Brown *et al*. [14] and Camprasse *et al*. [27] observed wildlife rescue hotspots associated with high population densities in southeast Queensland and Greater Melbourne, respectively. Further, given the large size of this dataset, it is likely these rescue records reflect an accurate trend in human–wildlife interactions in Greater Sydney. However, these data were not obtained via systematic repeated surveys or using rigorous scientific methods. Although we deleted obvious misidentifications to ensure information was as accurate as possible, the lack of standardized sampling should be considered when interpreting these results. Nonetheless, the identification of ‘rescue hotspots’ using wildlife rescue data could facilitate the creation of targeted scientific surveys and management strategies to protect wildlife.

In summary, wildlife rescue datasets capture large amounts of information over temporal and spatial scales that are almost impossible to replicate in targeted surveys. For snakes and lizards, taxa often overlooked in both research and conservation efforts, these datasets provide an opportunity to explore human–wildlife interactions. In the Greater Sydney region, there are clear compositional trends in the species being rescued and biases exhibited by humans towards certain species. Wildlife rescue datasets can help to inform species management practices, especially in conjunction with traditional survey techniques. Despite the biases and limitations present in such datasets, we need to find effective ways to utilize the massive amounts of information captured by volunteer wildlife rehabilitators. As urbanization increasingly impacts wildlife, human–wildlife interactions will become more common. Conservation biologists and managers would be wise to incorporate insights from wildlife rescue data to inform future management and monitoring strategies.

## Data Availability

Summary data tables available as supplementary material [48]. The full dataset is available from the Dryad Digital Repository [49].
